# Reorientation dynamics and ion diffusivity of neat dimethylimidazolium dimethylphosphate probed by NMR spectroscopy†

**DOI:** 10.1039/c9ra07731f

**Published:** 2019-11-04

**Authors:** Christoph Wiedemann, Günter Hempel, Frank Bordusa

**Affiliations:** Institute of Biochemistry and Biotechnology, Charles Tanford Protein Center, Martin Luther University Halle-Wittenberg Kurt-Mothes-Str. 3a D-06120 Halle (Saale) Germany christoph.wiedemann@biochemtech.uni-halle.de frank.bordusa@biochemtech.uni-halle.de; Institute of Physics, Martin Luther University Halle-Wittenberg Betty-Heimann-Str. 7 D-06120 Halle (Saale) Germany

## Abstract

NMR spectroscopy at two magnetic field strengths was employed to investigate the dynamics of dimethylimidazolium dimethylphosphate ([C_1_C_1_IM][(CH_3_)_2_PO_4_]). [C_1_C_1_IM][(CH_3_)_2_PO_4_] is a low-melting, halogen-free ionic liquid comprising of only methyl groups. ^13^C spin–lattice relaxation rates as well as self-diffusion coefficients were measured for [C_1_C_1_IM][(CH_3_)_2_PO_4_] as a function of temperature. The rotational correlation times, *τ*_c_, for the cation and the anion were obtained from the ^13^C spin–lattice relaxation rates. Although from a theoretical point of view cations and anions are similar in size, they show different reorientation mobilities and diffusivities. The self-diffusion coefficients and the rotational correlation times were related to the radii of the diffusing spheres. The analysis reveals that the radii of the cation and the anion, respectively, are different from each other but constant at temperatures ranging from 293 to 353 K. The experimental results are rationalised by a discrete and individual cation and anion diffusion. The [(CH_3_)_2_PO_4_]^−^ anion reorients faster compared to the cation but diffuses significantly slower indicating the formation of anionic aggregates. Relaxation data were acquired with standard liquid and magic-angle-spinning NMR probes to estimate residual dipolar interactions, chemical shift anisotropy or differences in magnetic susceptibility within the sample.

## Abstract 2

Liquid and HR-MAS NMR spectroscopy at two magnetic field strengths was employed to investigate the dynamics of ([C_1_C_1_IM][(CH_3_)_2_PO_4_]).

## Graphical abstract

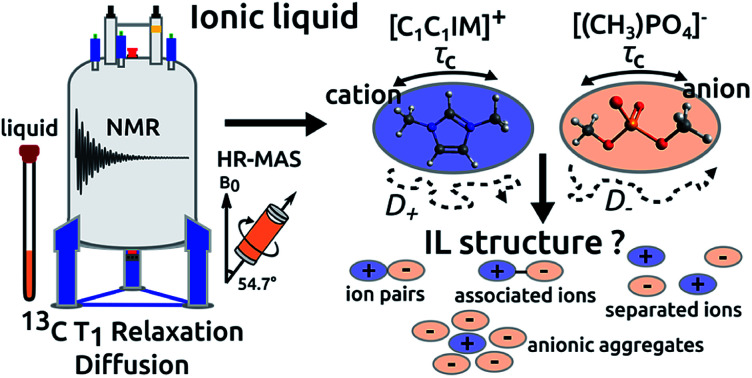

## Introduction

Organic salts with a melting point at ambient temperatures (melting points below 373 K) are commonly referred to as ionic liquids (ILs).^1,2^ ILs consist almost exclusively of organic cations and in-/organic anions. They are generally characterised by physical and chemical properties different from other molecular or atomic solvents, *e.g.* negligible vapour pressure, high chemical and thermal stability, high ionic conductivity and non-flammability. Because of their physicochemical characteristics, ILs have attracted wide attention as promising environmental friendly, “green” alternatives for commonly used organic solvents. The possibility of fine-tuning IL properties by an enormous diversity of cation–anion combinations renders ILs as substitute solvents or solvent additives in a wide range of laboratory and industrial applications.^1,3–6^ In addition to application in chemistry and physics, ILs have attracted considerable attention as solvents or solvent-mixtures in biochemistry.^7–12^

A deeper understanding of IL properties at a molecular or even atomic level is of vital interest with respect to the rational design of novel ILs or the previous knowledge about the suitability of ILs for a desired process. Facing the plethora of various types and classes of ILs, a comprehensive characterization of the physicochemical properties is, realistically seen, only possible for selected cation–anion combinations. The prediction of the physicochemical characteristics, and, maybe even more importantly, the potential performance for novel task-driven ILs based on structure–function relationships of known cation–anion combinations is a great challenge. Hence, a combined approach integrating spectroscopic, experimental and theoretical/computational methods is required to broaden our understanding of ILs and the cationic–anionic interplay among each other and with solutes as well.

Nuclear magnetic resonance (NMR) is a powerful spectroscopic techniques for studying compounds or molecular systems at an atomic level. Despite some experimental limitations (*e.g.* high IL viscosity, radio frequency absorption due to high concentration of charged particles resulting in sample heating, detuned frequency channels, or with respect to solutes the suppression of IL solvent signals) it has been frequently shown that ILs can be thoroughly investigated by NMR.^13–18^ By means of NMR spectroscopy different types of information on IL structure and dynamics are readily accessible by probing chemical shifts, nuclear Overhauser effects (NOEs), relaxation times or self-diffusion coefficients. The atomic composition of ILs offers an intrinsic set of NMR active spin-1/2 nuclei, such as ^1^H, ^13^C, ^15^N, ^19^F, ^31^P, suitable for investigation. In order to understand IL properties as a whole and the single contributions of the IL cation and anion respectively, to the observable IL properties, the characterization of the molecular dynamics is of great interest. The relaxation properties and diffusivity of selected imidazolium-based ILs have been successfully investigated recently using NMR spectroscopy.^19–28^

**Fig. 1:**
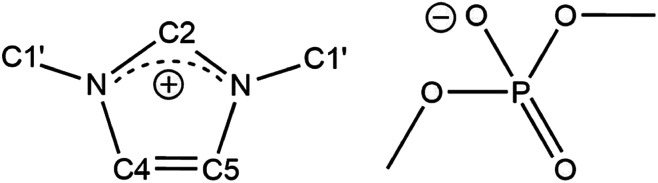
Chemical structure and denotation of dimethylimidazolium dimethylphosphate ([C_1_C_1_IM][(CH_3_)_2_PO_4_]).

Here, we examine and analyse ^13^C spin–lattice relaxation times (*T*_1_, relaxation rate: *R*_1_ = 1/*T*_1_) as well as self-diffusion coefficients of the ionic liquid dimethylimidazolium dimethylphosphate ([C_1_C_1_IM][(CH_3_)_2_PO_4_], Fig. 1) over a wide temperature range. In contrast to other imidazolium-based ILs, [C_1_C_1_IM][(CH_3_)_2_PO_4_] is a low-melting (liquid at room temperature), halogen-free IL comprising only methyl groups. The relaxation data were acquired with standard liquid probes at two magnetic field strengths and compared with data collected with high-resolution magic-angle spinning (HR-MAS) probes.

## Materials and methods

### Samples

[C_1_C_1_IM][(CH_3_)_2_PO_4_] was purchased from IoLiTec GmbH (Heilbronn, Germany) in highest available purity (≥98%) and used without further purification. The purity was confirmed by ^1^H and ^13^C NMR spectroscopy. The ^1^H and ^13^C NMR spectra [C_1_C_1_IM][(CH_3_)_2_PO_4_] are depicted in Fig. S1 and S2 (ESI†). Due to molecular symmetry the chemical shifts of H4/C4 and H5/C5 are respectively equivalent. For each ^1^H and ^13^C species of [C_1_C_1_IM][(CH_3_)_2_PO_4_] only single resonances were observed. No signals indicating impurities could be observed in the ^1^H spectrum except a very weak water peak. The water peak intensity was in the range of ^13^C satellite signal intensity of [C_1_C_1_IM][(CH_3_)_2_PO_4_] ^1^H resonances and originated from H_2_O traces in the used D_2_O.

### NMR measurements

All NMR experiments were acquired on Bruker AvanceIII systems with different magnetic field strengths, 9.35 T and 16.45 T, corresponding to a ^13^C resonance frequency of 100.6 MHz and 176.2 MHz, respectively. Both systems were equipped with 5 mm room-temperature liquid probes and 4 mm triple resonance HR-MAS probes. Liquid probe samples were fitted with a coaxial insert containing D_2_O for field frequency locking. All HR-MAS NMR experiments were collected without field frequency locking and the sample spin rate was 6 kHz. The field drift of both magnets was less than 0.3 Hz per hour without field frequency locking. The sample temperatures were controlled by variable temperature units. Temperature calibration was carried out with 4% MeOH in CD_3_OD for the low temperature range (278.2–300.2 K) and 80% ethylene glycol in DMSO-d_6_ for the high temperature range (300.2–353.2 K).

Inversion recovery experiments (180°–*τ*–90°) with power gated ^1^H decoupling were collected and the ^13^C longitudinal relaxation times (*T*_1_) were calculated from signal heights by a single exponential fit. For all experiments the relaxation delay was at least five times the longitudinal relaxation time of the slowest relaxing nucleus.

Diffusion coefficients of [C_1_C_1_IM][(CH_3_)_2_PO_4_] were measured at several temperatures using the double stimulated echo bipolar pulse-gradient pulse sequence (dstebpgp3s) for convection compensation and longitudinal eddy current delay implemented in the standard Bruker pulse library. The experimental signal amplitudes *S* were fitted to the Stejskal–Tanner equation^29,30^*S*/*S*_0_ = exp[−*γ*^2^*δ*^2^*g*^2^(*Δ* − *δ*/3)*D*]. *γ* is the ^1^H gyromagnetic ratio, *δ* holds the gradient pulse length, *g* is the gradient strength, *Δ* reflects the diffusion time and *D*_t_ is the diffusion coefficient. *δ* was fixed at 3 ms, and *Δ* was set appropriately. In order to avoid signal attenuation caused by the ^1^H *T*_1_ relaxation timing parameters were kept constant and only the gradient strength *g* was varied in 32 linear steps from 2 to 95% of the maximum probe gradient strength (4.78 G mm^−1^). The probe gradient system was calibrated by measuring the diffusion coefficient of a water sample at 298.2 K and compared with the literature value (*D*_t_ = 2.299 × 10^−9^ m^2^ s^−1^).^31^

Data processing was performed with Topspin 3.5.6 (Bruker Biospin GmbH, Rheinstetten) and the relaxation data were analysed with the software Dynamics Center 2.4.4 (Bruker Biospin GmbH, Rheinstetten).

### Theoretical background

For any given system, the observed longitudinal relaxation rates are in general caused by a combination of different relaxation mechanisms and can be expressed in total according to eqn (1):1
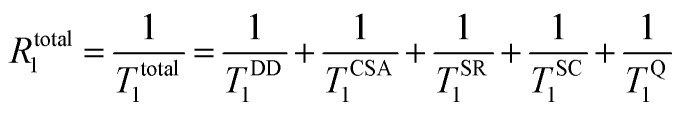
Typical relaxation mechanisms for nuclear spin systems are magnetic dipole–dipole (DD) interactions, interactions by anisotropy of chemical shifts (CSA), spin-rotation (SR), scalar coupling (SC) and electric quadrupoles (Q). The potential contributions from spin-rotations, scalar couplings or electric quadrupole interactions are either absent or negligible for spin-1/2 nuclei. Relaxation studies of heteronuclei, such as ^13^C, are long established and generally preferred to ^1^H.^32–35^ The relaxation properties for ^1^H mainly depend on inter- and intramolecular dipole–dipole interactions with surrounding protons. In contrast, ^13^C relaxation in most organic molecules usually results only from intramolecular dipolar coupling to directly attached protons. For proton-attached ^13^C nuclei intermolecular and contributions from not directly bonded protons can be safely ignored due to the strong ^1^H–^13^C distance dependence on *r*^−6^_H–C_.

The Bloembergen–Purcell–Pound (BPP) theory, first introduced for dipolar ^1^H relaxation^36^ and later extended to other heteronuclei including ^13^C,^37^ provides the theoretical basis for describing the observed *T*_1_ temperature dependence in terms of rotational correlation time and resonance frequency. Under broadband ^1^H decoupling and neglecting cross-correlation effects between different interactions, the dipolar longitudinal relaxation rate of proton-attached ^13^C nuclei is given by eqn (2).2
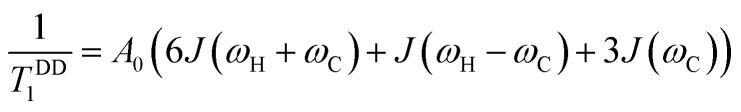
*J*(*ω*) are the spectral densities with the resonance frequencies, *ω*_H_ and *ω*_C_, of ^1^H and ^13^C. The constant *A*_0_ is defined by the number (*n*) of protons attached to ^13^C and given in brackets, the square of the dipole–dipole coupling constant which describes the magnitude of this coupling (eqn (3)). Here, *μ*_0_ is the vacuum permeability, *γ*_C_ and *γ*_H_ are the magnetogyric ratios of ^13^C and ^1^H nuclei, *ħ* is the reduced Planck constant and *r*_CH_ corresponds to the length of the C–H bond vector (1.09 Å).^34^ However, one should keep in mind that the actual C–H bond length can be a potential source of error in the calculation of rotational correlation times.^38^3
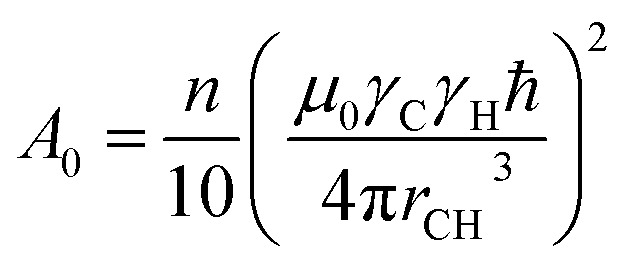
In summary, *A*_0_ is independent of temperature and frequency and takes the value of 2.15 × 10^9^ s^−2^ for *n* = 1 (6.45 × 10^9^ s^−2^ for *n* = 3).

Another source of ^13^C relaxation is CSA. For an axially-symmetric molecule, the principal components of the chemical shift tensor are parallel (*δ*_∥_) and perpendicular (*δ*_⊥_) to the symmetry axis and their difference (Δ*δ*) is relevant for relaxation. The longitudinal ^13^C relaxation due to CSA is given by eqn (4).4
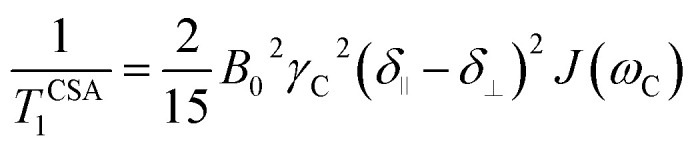


For proton-attached ^13^C nuclei with only moderately small CSA the contribution of CSA to the overall relaxation rate is one order of magnitude smaller than those for dipole–dipole relaxation and mostly neglected in the discussion of proton-attached ^13^C relaxation. However, the contribution of CSA to ^13^C longitudinal relaxation should be taken into account in particular at high magnetic fields and in situations where the nuclei under investigation exhibits large chemical shift ranges. In such a case, the total longitudinal relaxation rate for a proton-attached ^13^C nuclei is the sum of the rate due to dipolar interaction and CSA (eqn (5)).^39^5
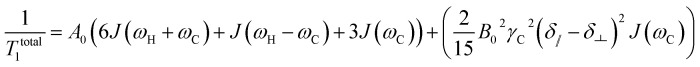


For rigid molecules with isotropic diffusion and a single molecular rotational correlation time (*τ*_c_) the spectral density *J*(*ω*) can be modelled by eqn (6):6
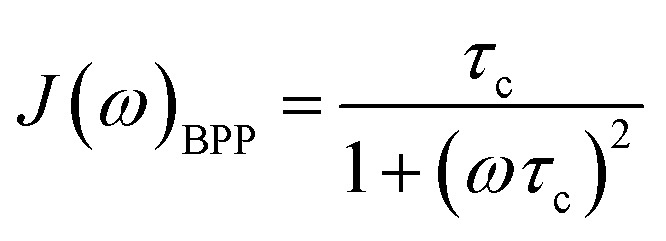


In situations where the ^1^H–^13^C dipolar interaction (eqn (2)) is the main source of ^13^C relaxation and the contribution of CSA is absent or not considered (second term in eqn (5) vanishes) it is worthwhile to note that eqn (2) by applying the BPP spectral density function (eqn (6)) reaches its maximum point, and hence *T*_1_ a minimum, at *ω*_C_*τ*_c_ = 0.791. Using the relation *ω*_H_/*ω*_C_ ≈ 3.98 and rearranging eqn (2) the theoretical ^13^C *T*_1_ minimum can be calculated by eqn (7):7
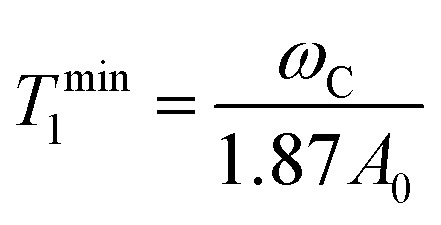


To account for contributions from additional intramolecular motion to relaxation the generalized order parameter *S*^2^ (0 < *S*^2^ ≤ 1) and an effective internal correlation time (*τ*_i_) were introduced in the “simple model-free” approach^40^ (eqn (8)).8
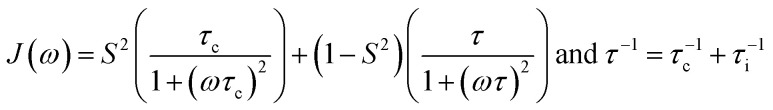
*S*^2^ gives a measure of the spatial restriction of intramolecular motion. An order parameter of *S*^2^ = 1 implies a complete rigid internuclear vector tumbling with the overall molecular correlation time *τ*_c_. *S*^2^ would approach 0 for complete unrestricted isotropic internal motion of the bond-vector.^39^

For *S*^2^ = 1 or in the slow intramolecular motion regime (*τ*_i_ ≫ *τ*_c_) eqn (8) reduces to eqn (6). In the fast intramolecular motion limit (*τ*_i_ ≪ *τ*_c_) eqn (8) reduces to *J*(*ω*) = *S*^2^*J*(*ω*)_BPP_. One elegant way to extract information about molecular mobility from ^13^C longitudinal relaxation times under the assumption of fast intramolecular motion and neglecting the CSA contribution is given in great detail in recent publications by Matveev *et al.*^41–43^ With the knowledge of the precise ^13^C *T*_1_ minimum it is possible to independently simplify the determination of the value of *S*^2^ (*S*^2^ = *ω*_C_/(1.87*A*_0_*T*^min^_1_)) and hence to calculate *τ*_c_ for any given *T*_1_.

Sometimes the relaxation of viscous liquids, even far above the melting point, is insufficiently described by eqn (6) or (8) and a correlation time distribution should be included into *J*(*ω*). For such systems the molecular motion and relaxation properties can be described more efficiently by a distribution of correlation times rather than a single correlation time and an order parameter.^44–48^ Therefore, the empirical Cole–Davidson (CD) approach^49,50^ (eqn (9)) is widely used in literature for quantitative analysis of relaxation data with distributed correlation times. In eqn (9) the parameter *β* (0 < *β* ≤ 1) describes the width of the distribution and *τ*_CD_ is related to *τ*_c_ by *τ*_c_ = *βτ*_CD_. For *β* = 1 eqn (9) simplifies to eqn (6).9
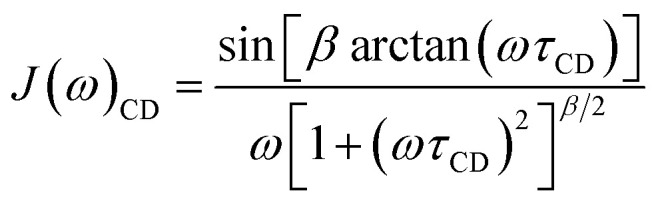
Eqn (9) can also be extended by *S*^2^ and *τ*_i_ to accomodate additional internal motion. For fast internal motion the spectral density is given by *J*(*ω*) = *S*^2^*J*(*ω*)_CD_.

In situations where a molecule undergoes anisotropic tumbling or has moieties with fast internal reorientation compared with the overall molecular motion (*e.g.* fast rotating methyl groups) the order parameter *S*^2^ can be replaced by the expression *S*_i_^2^ = 1/4(3 cos^2^(*φ*) − 1)^2^. Here, the motion of a ^13^C–^1^H vector around its rotation axis (symmetry axis) and the azimuthal angle *φ* is taken into account.^23,35,51–54^ For a methyl group with tetrahedral geometry (*φ* = 109.5°) *S*_i_^2^ takes the value 0.11. The motion of the methyl rotation axis itself is considered by setting *S*^2^ = 0.11*S*_Met_^2^.

It is generally accepted that the temperature dependence of *τ*_c_ follows the Arrhenius form (eqn (10)). *E*_A_ is the activation energy for rotational diffusion and *R* the universal gas constant.

10 *τ*_c_ = *τ*_0_e^*E*_A_/*RT*^

However, sometimes eqn (10) is only applied for fast internal motion and the overall correlation time is better described by a Vogel–Fulcher–Tammann (VFT) behaviour^2,23^ (eqn (11)).

11 *τ*_c_ = *τ*_VFT_e^*E*_VFT_/*R*(*T* − *T*_0_)^

The parameter *T*_0_ is in the order of the glass transition temperature and *E*_VFT_ is an apparent activation energy.

The experimental relaxation data for one ^13^C nucleus at both magnetic field strengths were simultaneously fitted with different models and parameter sets. Constrained least-square fitting was performed using Python scripts written in-house. Bounds *T*_0_ > 0, 0 < *S*^2^ ≤1 and 0< *β* ≤ 1 were imposed. The reduced *χ*^2^ value (*χ*_red_^2^) was used to assess the validity of the fits.

All mechanisms that contribute to ^13^C relaxation mainly arise from intramolecular contributions. Therefore, longitudinal ^13^C relaxation times are a reliable probe of molecular mobility and their analysis renders possible the characterization of molecular mobility in detail.

## Results

### Comparison HR-MAS *vs.* liquid probe

Most of the ^13^C relaxation studies rely on the assumption that after inversion the recovery of the longitudinal part of the magnetisation follows a single exponential process. However, in situations where ^13^C nuclei are directly attached to a proton, heteronuclear cross-relaxation by dipole–dipole interaction can lead to a non-exponential recovery of ^13^C longitudinal magnetisation unless the ^13^C spectra are collected with broadband ^1^H decoupling.^55^ The impact of cross-relaxation effects on the ^13^C–^1^H dipolar relaxation mechanism was estimated by determining the ^13^C *T*_1_ values under ^1^H broadband decoupling conditions enabling NOE enhancement and under ^1^H inverse gated decoupling without NOE enhancement at different magnetic fields and for selected temperatures (Table S1†). There are no or virtually negligible differences between the ^13^C *T*_1_ values measured under broadband and gated ^1^H decoupling at selected field strength and temperature. Based on these data we conclude that there is no impact of cross-relaxation in the ^13^C–^1^H dipole–dipole relaxation mechanism neither for the [C_1_C_1_IM]^+^ cation nor the [(CH_3_)_2_PO_4_]^−^ anion within the observed temperature range. The ^13^C relaxation by dipole–dipole interaction within [C_1_C_1_IM][(CH_3_)_2_PO_4_] can be considered as a single exponential process. This is in agreement with the result presented by Imanari *et al.*^19^ for the [C_3_C_1_IM]^+^ cation in [C_3_C_1_IM][Br].

To reduce residual dipolar interactions or differences in magnetic susceptibility that might be present in the sample, [C_1_C_1_IM][(CH_3_)_2_PO_4_] was also studied by HR-MAS NMR. As already shown for the high-resolution liquid probes even under magic-angle spinning there is nor difference in the ^13^C longitudinal relaxation times determined with broadband or gated ^1^H decoupling (Table S1†). The ^13^C *T*_1_ values of single carbons obtained by collecting spectra with standard liquid probes and HR-MAS probes are highly comparable at selected field strength and temperature. On one hand this reflects the accuracy of probe temperature calibration. On the other hand the orientational components (3 cos^2^ *θ* − 1) of the Hamiltonians for the dipolar interaction, CSA or differences in the magnetic susceptibility due to sample inhomogeneity are averaged to zero by molecular motion without additional sample spinning. Despite the high viscosity of [C_1_C_1_IM][(CH_3_)_2_PO_4_], the motional averaging is fast enough to remove the contributions from interactions which would lead to line broadening. This assumption is further confirmed by measuring the ^13^C line widths at several temperatures with high-resolution liquid and HR-MAS probes with 6 kHz sample spinning rate (Table S2†). There are no differences in the ^13^C line widths for each carbon at selected temperatures regardless which probe was used.

All ^13^C signals of [C_1_C_1_IM][(CH_3_)_2_PO_4_] are attenuated with decreasing temperature due to increasing line broadening, which indicates a restriction in the molecular motion. However, the methyl groups of the [C_1_C_1_IM]^+^ cation and the [(CH_3_)_2_PO_4_]^−^ anion are less attenuated compared with the cation ring carbons. This indicates a less restricted motion of the cationic as well as the anionic methyl groups compared with cation ring carbons.

In conclusion, for [C_1_C_1_IM][(CH_3_)_2_PO_4_] a sample spinning at the “magic angle” (*θ* = 54.7°) is not essential to improve the spectral resolution.

### ^13^C relaxation studies

The ^13^C longitudinal relaxation times of [C_1_C_1_IM][(CH_3_)_2_PO_4_] were measured at two magnetic field strengths under broadband proton decoupling within a temperature range from 278.2 K to 353.2 K in the next step. Based on this temperature dependence and the application of different models, characteristic rotational correlation times (*τ*_c_) were derived for each ^13^C nuclei in the molecule.

**Fig. 2:**
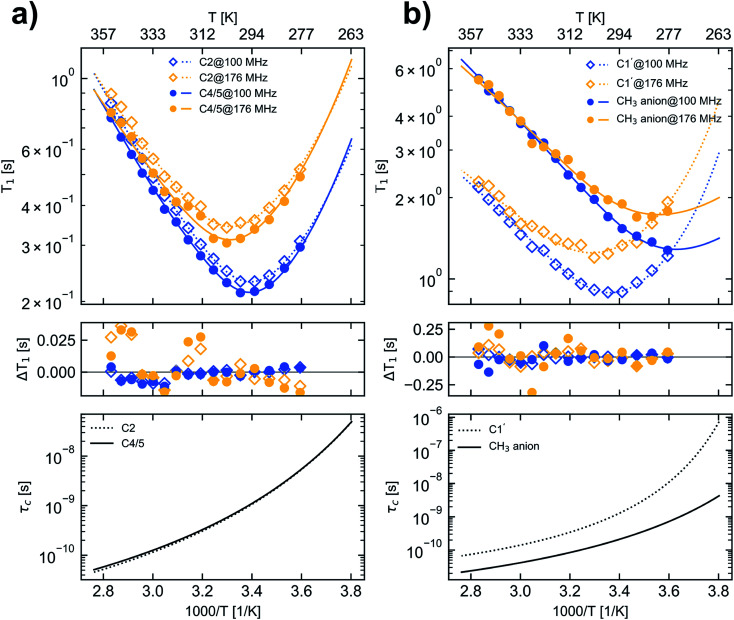
^13^C longitudinal relaxation times (upper panels), the difference between measured and fitted relaxation times (middle panel) and the calculated *τ*_c_ values (lower panel) for carbons in [C_1_C_1_IM][(CH_3_)_2_PO_4_] as a function of temperature. (a) carbon C2 (open squares and dotted lines) and C4/5 (filled circles and solid lines), (b) carbon C1′ (open squares and dotted lines) and CH_3_ carbons of the anion (filled circles and solid lines). For denotation see Fig. 1. Blue and yellow markers represent data measured at *B*_0_ of 9.35 T and 16.45 T corresponding to a ^13^C resonance frequency of 100.6 MHz and 176.2 MHz, respectively. The lines in the upper panel represent calculated ^13^C relaxation times according the fit parameters given in Table 2D.

The temperature dependence of the ^13^C longitudinal relaxation times is shown in Fig. 2. At first, with increasing temperature the magnitude of the ^13^C *T*_1_ values decreases for all cation carbons until reaching a minimum. After passing the minimum the magnitude of the ^13^C *T*_1_ values increase with increasing temperature. At a field strength of 9.35 T C2, C4/5 and C1′ of the imidazolium cation show a *T*_1_ minimum at approx. 298 K. For C2 and C4/5 the observed *T*_1_ minima of 0.231 s and 0.213 s, respectively, are close to the theoretical *T*_1_ minimum of 0.158 s for pure dipole–dipole relaxation of a CH group at that field strength assuming a BPP spectral density function (eqn (7)). At 16.45 T the *T*_1_ minimum of C2, C4/5 and C1′ is uniformly shifted to 303 K and for C2 and C4/5 the values in the *T*_1_ minimum (0.341 s and 0.305 s) closely match the calculated *T*_1_ minimum of 0.276 s. The fact that all [C_1_C_1_IM]^+^ cation ring carbons for a given magnetic field strength have a *T*_1_ minimum at nearly the same temperature indicates an isotropic reorientation of the cation. The deviation between the measured and the calculated relaxation times can be explained by a distribution of correlation times or additional internal motion of the H–C bond vector, which corroborates the application of “model-free” approach (eqn (8)).

At all temperatures the product of the *T*_1_ relaxation times of the C1′ methyl nuclei and the number of attached protons (*n* = 3) is larger than those of the C2 and C4/5 CH groups (*n* = 1) by a factor of 9–12. The inverse of this factor is in the order of 0.1 corresponding to the aforementioned expression of *S*^*2*^ for fast rotating methyl groups with tetrahedral geometry. Furthermore, the measured C1′ relaxation times indicate an unrestrained rotational motion of the [C_1_C_1_IM]^+^ methyl groups. The theoretical ^13^C *T*_1_ values for dipole–dipole relaxation of a methyl group with free internal rotation also depend on the selected X–C–H bond angle *φ*. For *φ* = 109.5° ^13^C *T*_1_ of a rapidly rotating CH_3_ group which is 3-times longer than for a CH moiety under the assumption that both have the same effective rotational correlation time. With an increasing bond angle (*φ* > 109.5°) the longitudinal ^13^C relaxation time of CH_3_ theoretically increases further by a factor of ≈4, ≈4.5 and ≈5 for *φ* values of 112°, 113° and 114°, respectively, compared to a CH group. A rough estimation of the ^13^C *T*_1_ ratio of C1′ and C2 or C4/5 of the [C_1_C_1_IM]^+^ cation suggests that the bond angle *φ* of the methyl group is slightly greater than 109.5°.

The methyl carbons in the [(CH_3_)_2_PO_4_]^−^ anion only reveal a *T*_1_ minimum at 288 K for a field strength of 16.45 T, indicating a much more active motion compared with the carbons of the cation. Within the selected temperature range no *T*_1_ minimum could be observed at 9.35 T for the methyl carbons of the [(CH_3_)_2_PO_4_]^−^ anion. It can be assumed that the methyl carbon of the [(CH_3_)_2_PO_4_]^−^ anion would also show a minimum in the ^13^C *T*_1_ relaxation time at 9.35 T, which however would appear only at a lower temperature than accessible in this study. For the methyl carbons in the [(CH_3_)_2_PO_4_]^−^ anion no clear field strength dependence of the *T*_1_ relaxation could be observed in the high temperature range (>320 K). Also for the carbons of the cation the differences in the *T*_1_ values reduce with increasing temperatures (Fig. 2, Table S3†).

### CSA contribution to ^13^C relaxation

Mostly, the relaxation of proton attached ^13^C nuclei in the liquid state is solely discussed in terms of dipolar interaction and the contribution of CSA to relaxation is neglected in literature.

Measuring ^13^C *T*_1_ relaxation times at different magnetic field strengths in the fast motion limit (*ω*_*C*_*τ*_c_ ≪ 1) directly allows an estimation of the CSA contribution to relaxation. From the data obtained in this study, we can not completely exclude CSA contributions to relaxation for any carbons of [C_1_C_1_IM][(CH_3_)_2_PO_4_] but the comparable ^13^C *T*_1_ times at different magnetic field strengths in the high temperature range indicate only a minor effect of CSA to relaxation compared to dipole–dipole interaction. This is in agreement with the observation of Imanari *et al.*^19^ who stated also a minor impact of CSA for imidazolium based IL cations. However, in situations where CSA additionally contributes to ^13^C relaxation eqn (5) should be more suitable to represent the measured ^13^C *T*_1_ relaxation times. Therefore, we analysed our data in two ways: without taking account of CSA and in consideration of CSA.

### Calculation of *τ*_c_ and *E*_a_

Considering the above remarks, at first we treated the ^13^C relaxation times of [C_1_C_1_IM][(CH_3_)_2_PO_4_] as a result of the dipole–dipole relaxation mechanism between carbons and directly attached protons which can be described readily by eqn (2) and the modified BPP spectral density function (eqn (8)). We assume that the intramolecular motion is at least one order of magnitude faster than the overall molecular motion (*τ*_*i*_≪*τ*_c_) so that the second term in eqn (8) is negligible. For comparison the CD spectral density (eqn (9)) was also used for fitting. The temperature dependence of *τ*_c_ was modelled with the Arrhenius (eqn (10)) and VFT (eqn (11)) approach, respectively. In the Arrhenius approach one activation energy is assumed for the entire temperature range. However, for viscous liquids a decreasing temperature can correlate with an increasing activation energy. Such a behaviour is sometimes better described by the VFT equation.^2,23^ For [C_1_C_1_IM][(CH_3_)_2_PO_4_] the precise glass transition temperature is not known but it was experimentally shown that for imidazolium based ILs the glass transition temperature is in the range of 180–220 K.^56–61^ The fit parameters are summarized in Table 1.

Fit parameter values for the ^13^C relaxation expressed by eqn (2). The spectral density functions given in eqn (8) (A, C) and eqn (9) (B, D) were used for fitting. The temperature dependence of *τ*_c_ was fitted with eqn (10) (A, B) and eqn (11) (C, D), respectively

**Table:** 

		*S* ^2^	*E*_A_ (kJ mol^−1^)	*τ*_0_ (s)	*τ*_c_ at 298 K (ns)	*χ*_red_^2^
(A)	C2	0.70 (0.87^d^)	38.18	1.67 × 10^−16^	0.82	2.98^b^/6.78^c^
	C4/5	0.76	38.27	1.67 × 10^−16^	0.85	3.60^b^/8.26^c^
	C1′	0.56^a^	32.22	2.09 × 10^−15^	0.93	4.87^b^/12.34^c^
	CH_3_ anion	0.64^a^	18.97	6.58 × 10^−14^	0.14	1.06^b^/1.36^c^

a Fast methyl-group rotation was considered by modifying *S*^2^ with the factor 0.11 (*S*^2^ = 0.11*S*_Met_^2^). For methyl carbons *S*_Met_^2^ is given.

b Reduced *χ*^2^ values for data at 9.35 T.

c Reduced *χ*^2^ values for data at 16.45 T.

d Fitted *S*^2^ with a C–H bond length of 1.13 Å.

**Table:** 

		*β*	*S* ^2^	*E*_A_ (kJ mol^−1^)	*τ*_0_ (s)	*τ*_CD_/*τ*_c_ at 298 K (ns)	*χ*_red_^2^
(B)	C2	0.48	0.92	41.89	7.65 × 10^−17^	1.68/0.80	2.83^b^/6.86^c^
	C4/5	0.50	0.97	41.72	8.13 × 10^−17^	1.67/0.83	3.45^b^/8.39^c^
	C1′	0.42	0.78^a^	36.59	8.61 × 10^−16^	2.23/0.93	4.29^b^/13.26^c^
	CH_3_ anion	0.44	1.00^a^	19.06	9.39 × 10^−14^	0.20/0.09	1.33^b^/1.60^c^

**Table:** 

		*S* ^2^	*E*_VFT_ (kJ mol^−1^)	*T*_0_ (K)	*τ*_0_ (s)	*τ*_c_ at 298 K (ns)	*χ*_red_^2^
(C)	C2	0.72 (0.89^d^)	8.33	165.94	3.81 × 10^−13^	0.76	1.93^b^/7.21^c^
	C4/5	0.78	6.89	179.09	7.24 × 10^−13^	0.77	2.41^b^/8.51^c^
	C1′	0.58^a^	3.91	203.34	5.72 × 10^−12^	0.82	4.99^b^/12.53^c^
	CH_3_ anion	—	—	—	—	—	—

**Table:** 

		*β*	*S* ^2^	*E*_VFT_ (kJ mol^−1^)	*T*_0_ (K)	*τ*_0_ (s)	*τ*_CD_/*τ*_c_ at 298 K (ns)	*χ*_red_^2^
(D)	C2	0.43	1.00	5.16	203.93	2.26 × 10^−12^	1.66/0.71	1.21^b^/5.47^c^
	C4/5	0.51	1.00	4.96	204.40	2.47 × 10^−12^	1.46/0.74	1.62^b^/6.82^c^
	C1′	0.29	1.00^a^	2.19	238.76	3.03 × 10^−11^	2.64/0.76	3.74^b^/9.89^c^
	CH_3_ anion	—	—	—	—	—	—	—

Regardless of the type of spectral density function or temperature dependence which is applied, all fits provide comparable results with respect to the order parameter, activation energy and rotational correlation time. Even the obtained *T*_0_ parameters are in agreement with values reported in literature. There is no difference in the goodness-of-fit between the application of the Arrhenius or the VFT approximation for the temperature dependence of *τ*_c_. The same applies for the used spectral density functions. However no reliable fit could be obtained for the methyl carbon of the anion by applying the VFT approach for the *τ*_c_ temperature dependence. It has to be noted that the application of the Arrhenius approach provides very good fits for the methyl carbon of the anion. The experimental data at 9.35 T are obviously better represented by the fit parameters than the relaxation data at 16.45 T potentially indicating a more pronounced CSA contribution at the higher field strength.

In the next step we treated the ^13^C relaxation as a combination of dipole–dipole interaction and CSA (eqn (5)) under the assumption of fast internal motion. Again the experimental data were fitted to a BPP (eqn (8)) and CD (eqn (9)) type spectral density function and the Arrhenius (eqn (10)) and VFT (eqn (11)) approach for the temperature dependence of *τ*_c_, respectively. The obtained fit parameters are compiled in Table 2. Taking account of CSA significantly improves the goodness-of-fit and the experimental data at both magnetic field strengths are well represented by the fit parameters. The estimated Δ*δ* values for the IL ring carbons are in the range of 112–142 ppm. This is in agreement with Δ*δ* for aromatic ring carbons reported by others.^23,62,63^ However, the fitted Δ*δ* values (Table 2) for the methyl carbons are unexpectedly high when the motion of the ^13^C–^1^H vector around its rotation axis is considered (*S*^2^ = 0.11*S*_Met_^2^). Excluding the factor 0.11 during fitting would result in Δ*δ* values in the range of 40–60 ppm for the methyl carbons.

Fit parameter values for the ^13^C relaxation expressed by eqn (5). The spectral density functions given in eqn (8) (A, C) and eqn (9) (B, D) were used for fitting. The temperature dependence of *τ*_c_ was fitted with eqn (10) and (11), respectively

**Table:** 

		*S* ^2^	*E*_A_ (kJ mol^−1^)	*τ*_0_ (s)	*τ*_c_ at 298 K (ns)	Δ*δ* (ppm)	*χ*_red_^2^
(A)	C2	0.60 (0.75^d^)	38.26	1.79 × 10^−16^	0.92	134.28	1.54^b^/1.39^c^
	C4/5	0.64	38.21	1.92 × 10^−16^	0.96	142.58	1.69^b^/1.77^c^
	C1′	0.45^a^	32.09	2.57 × 10^−15^	1.09	280.20	1.11^b^/2.03^c^
	CH_3_ anion	0.44^a^	22.99	2.14 × 10^−14^	0.23	207.06	0.82^b^/1.09^c^

a Fast methyl-group rotation was considered by modifying *S*^2^ with the factor 0.11 (*S*^2^ = 0.11*S*_Met_^2^). For methyl carbons *S*_Met_^2^ is given.

b Reduced *χ*^2^ values for data at 9.35 T.

c Reduced *χ*^2^ values for data at 16.45 T.

d Fitted *S*^2^ with a C–H bond length of 1.13 Å.

**Table:** 

		*β*	*S* ^2^	*E*_A_ (kJ mol^−1^)	*τ*_0_ (s)	*τ*_CD_/*τ*_c_ at 298 K (ns)	Δ*δ* (ppm)	*χ*_red_^2^
(B)	C2	0.60	0.72	40.89	1.02 × 10^−16^	1.50/0.90	112.64	1.68^b^/0.72^c^
	C4/5	0.65	0.74	40.43	1.18 × 10^−16^	1.45/0.94	121.37	1.85^b^/1.24^c^
	C1′	0.57	0.55^a^	34.97	1.39 × 10^−15^	1.89/1.07	280.08	0.95^b^/1.65^c^
	CH_3_ anion	0.27	0.64^a^	28.91	6.99 × 10^−15^	0.82/0.22	264.62	2.04^b^/1.07^c^

**Table:** 

		*S* ^2^	*E*_VFT_ (kJ mol^−1^)	*T*_0_ (K)	*τ*_0_ (s)	*τ*_c_ at 298 K (ns)	Δ*δ* (ppm)	*χ*_red_^2^
(C)	C2	0.61 (0.75^d^)	23.63	66.82	4.10 × 10^−15^	0.89	132.84	1.22^b^/1.74^c^
	C4/5	0.65	18.78	93.24	1.49 × 10^−14^	0.92	140.40	1.16^b^/2.24^c^
	C1′	0.45^a^	17.07	84.80	6.94 × 10^−14^	1.06	277.69	1.08^b^/2.35^c^
	CH_3_ anion	0.32^a^	4.29	191.29	3.01 × 10^−12^	0.38	279.79	0.65^b^/0.99^c^

**Table:** 

		*β*	*S* ^2^	*E*_VFT_ (kJ mol^−1^)	*T*_0_ (K)	*τ*_0_ (s)	*τ*_CD_/*τ*_c_ at 298 K (ns)	Δ*δ* (ppm)	*χ*_red_^2^
(D)	C2	0.33	1.00	5.54	203.36	2.05 × 10^−12^	2.37/0.78	125.80	0.08^b^/0.49^c^
	C4/5	0.37	1.00	5.26	205.03	2.44 × 10^−12^	2.20/0.81	134.09	0.13^b^/0.85^c^
	C1′	0.21	1.00^a^	2.62	236.28	2.61 × 10^−11^	4.35/0.90	267.22	0.31^b^/0.80^c^
	CH_3_ anion	0.12	1.00^a^	3.52	210.58	1.12 × 10^−11^	1.42/0.17	303.64	0.22^b^/1.13^c^

The calculated *τ*_c_, for example at 298 K, are nearly equal within the selected spectral density approach for all carbons of the [C_1_C_1_IM]^+^ cation characterizing a uniform reorientation and molecular mobility. Moreover, our ^13^C relaxation measurements at two different magnetic field strengths reveal nearly the same rotational correlation times for all cation carbons (Tables 1 and 2) regardless of the selected type of spectral density function or approach for the temperature dependence of *τ*_c_. Under this point of view the assumption of isotropic motion for the imidazolium ring is justified at least at room temperature. The activation energies (*E*_*A*_) of molecular reorientation for the imidazolium ring carbons are in the range of 32–38 kJ mol^−1^ for BPP type spectral density and 39–42 kJ mol^−1^ for CD approach, respectively. Using VFT approach to model the temperature dependence of *τ*_c_ results in activation energies (*E*_VFT_) within 2–8 kJ mol^−1^. These values are consistent with results obtained by others for imidazolium ring carbons and attached methyl groups.^19,23,41,42,64^ The fitted *E*_A_/*E*_VFT_ values are nearly unaffected by the inclusion of CSA to the analysis of the relaxation data. Only the combination of BPP spectral density function, including the CSA relaxation mechanism and a VFT type *τ*_c_ temperature dependency results in high *E*_VFT_ values of 17–23 kJ mol^−1^ and low *T*_0_ values for the [C_1_C_1_IM]^+^ carbons (Table 2C).

It was shown recently that the behaviour of the anion as a whole can be reasonably described by values obtained for methyl groups in carbon containing IL anions.^43,64^ For the methyl carbons of the anion, the calculated activation energies (*E*_A_/*E*_VFT_) are considerably lower compared with cation carbons. At 298 K *τ*_c_ is at least three to five times shorter than the corresponding values of the cation indicating a faster reorientation mobility of the anion. Moreover, this emphasises the hypothesis that cation and anion behave independently as dissociated ions and may form rather short-living ion pairs. We wish to point out that the molecular mobilities of [C_1_C_1_IM]^+^ and [(CH_3_)_2_PO_4_]^−^ should be considered as time-weighted averages between the reorientation dynamics of tightly associated [C_1_C_1_IM]–[(CH_3_)_2_PO_4_] ion pairs and single dissociated ions.

The S^2^ of the ring carbons C2 (≈0.6–0.72) and C4/5 (≈0.64–0.78) calculated under the assumption of a C–H bond length of 1.09 Å and a BPP type spectral density would reveal a moderate degree of additional internal motion. The slightly lower *S*^2^ of C2 compared to C4/5 suggests accordingly that the proton attached to C2 is not preferentially involved in H-bonding in comparison to the other ring protons. The participation of the cationic H2 in hydrogen bonding with anions should result in a more constrained orientation, and hence higher order parameter of the corresponding C–H vector. The prominent role of the C2 position of imidazolium based IL cations in interacting with anions mainly *via* hydrogen bonding is extensively described in literature.^18,23,65–71^ It is well known that the length of a C–H bond can vary depending on the hybridization of the carbon atom and the polarity of the bond.^72^ In this context, Antony *et al.*^73^ postulated for the strong hydrogen bonding donor at position C2 of the imidazolium based cation (in this case 1-butyl-3-methylimidazolium) a C–H bond length of 1.13 Å. Reevaluating the C2 data with an elongated C–H bond length results in higher *S*^2^ values. By applying a C–H bond length of 1.13 Å *S*^2^ of C2 increases to values up to 0.99. These higher order parameters would corroborate the hypothesis that the proton in C2 position acts in hydrogen bond formation with the anion also for [C_1_C_1_IM][(CH_3_)_2_PO_4_]. The H-bond formation mainly between the CH group in position 2 of the cation and the [(CH_3_)_2_PO_4_]^−^ anion is in agreement with existing hypothesis about the cationic–anionic interaction in imidazolium based ILs and the ability of [(CH_3_)_2_PO_4_]^−^ to act as relatively strong H-bond acceptor.^71^ The order parameters obtained for the methyl groups in C1′ and the anion are slightly smaller compared to the ring carbons when the BPP type spectral density is applied for fitting. This indicates a slightly higher flexibility of the methyl carbons.

With respect to *S*^2^ the results are somewhat different when the CD spectral density function is used. Here, the best fits were obtained when the fit parameter *S*^2^ takes rather high values which would correlate with no or only very limited internal motion. However, the low *β* values of all carbons in [C_1_C_1_IM][(CH_3_)_2_PO_4_] could point to a broad distribution of correlation times.

The consistency between the fit parameters and the data derived at two magnetic field strengths justifies the chosen theoretical models and the approach of evaluation. The combination of applying the CD type spectral density function, the VFT approach for the *τ*_c_ temperature dependence and taking CSA into account provides the best fit results for our experimental data within the selected temperature range (Fig. 2). Experimental data at lower temperatures or higher magnetic field strengths than accessible in this study would further improve the reliability of the proposed fitting approach mainly for the more mobile IL anion. However, we do not want to conceal that our experimental data are also considerably well represented by the simple assumption of dipolar relaxation and the BPP spectral density function including *S*^2^ only (Table 1A).

As already mentioned (see Theoretical background) in situations where the temperature dependence of the ^13^C *T*_1_ values reveals a precise minimum the independent calculation of *S*^2^ (*S*^2^ = *ω*_C_/(1.87*A*_0_*T*^min^_1_)) and thus *τ*_c_ for any given *T*_1_ is possible. For further information we refer to the recent publications by Matveev *et al.*^41,42,64^ Since the ^13^C nuclei in [C_1_C_1_IM][(CH_3_)_2_PO_4_] reveal *T*_1_ minima, we also calculated *S*^2^ and extracted *τ*_c_ values for each temperature for comparison as proposed by the authors. Finally the *τ*_c_ temperature dependence was fitted according the Arrhenius (eqn (10)) and VFT approach (eqn (11)). The motional characteristics of [C_1_C_1_IM][(CH_3_)_2_PO_4_] applying this approach are summarized in Table S4 and depicted in Fig. S3 and S4.† Particularly with respect to *S*^2^, *τ*_c_ and *E*_A_ the fit parameters obtained by applying the BPP spectral density function and neglecting any CSA contributions are in remarkable agreement with the values obtained by simultaneous fitting the relaxation data of two magnetic field strengths (Tables 1 and 2). In conclusion, if a clear temperature dependent ^13^C *T*_1_ minimum is observable at one magnetic field strength, the calculation of *S*^2^ in that point, and thereafter *τ*_c_ and *E*_A_, provides a robust and reliable approach for the extraction of information about molecular motion.

### Nuclear Overhauser effect

**Fig. 3:**
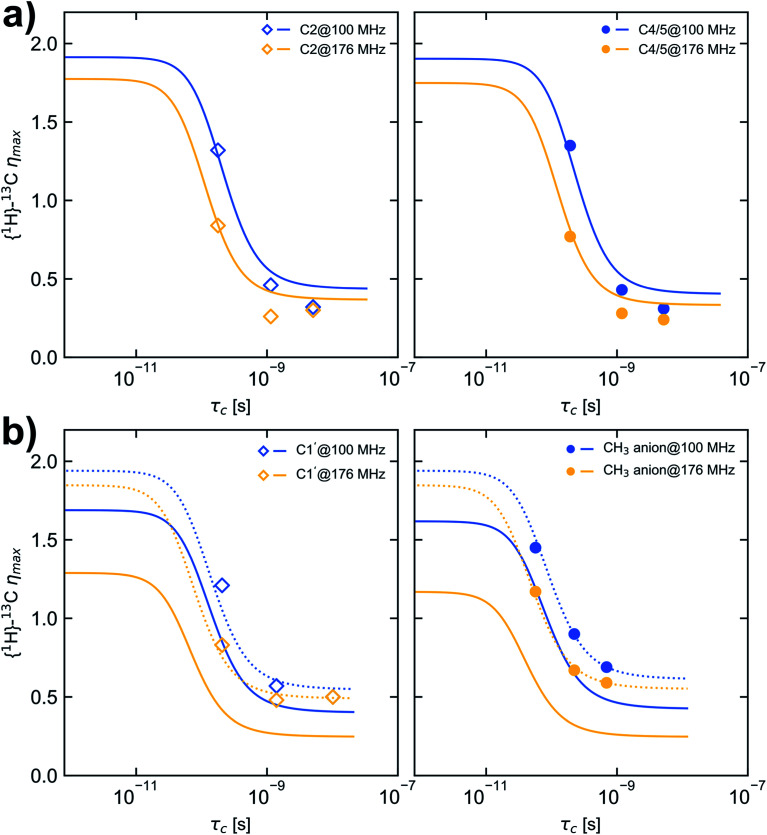
Dependence of maximum {^1^H}–^13^C NOE enhancement (*η*_max_) on *τ*_c_ calculated for a CD spectral density function and considering CSA with the fit parameters given in Table 2D (solid lines). (a) carbon C2 (left, open squares) and C4/5 (right, filled circles), (b) carbon C1′ (left, open squares) and CH_3_ carbon of the anion (right, filled circles). Dashed lines in (b) are calculated with the fit parameters given in Table 2D but a CSA value of 100 ppm. Blue and yellow markers represent data measured at *B*_0_ of 9.35 T and 16.45 T corresponding to a ^13^C resonance frequency of 100.6 MHz and 176.2 MHz, respectively.

The nuclear Overhauser effect (NOE) is an import consequence of dipolar relaxation. In addition, to validate that the ^13^C relaxation proceeds mainly by dipole–dipole interaction with attached protons the NOE enhancement factors *η* were measured in addition for selected temperatures, field strengths and probe settings (Table S5† and Fig. 3). Depending on the selected field strength comparable enhancement factors were obtained for each temperature using either HR-MAS or liquid probes.

The maximum *η* observable relies on the rotational correlation time and thus on the molecular motion of the nuclei under investigation. Other relaxation pathways than intramolecular dipole–dipole interaction, which contribute to the longitudinal relaxation rate (*e.g.* CSA or intermolecular dipole–dipole interaction), can reduce the maximum *η* (eqn (12)).^39^ Here we examine the ^13^C NOE enhancement taking CSA into account. In the case that CSA is not considered the term (Δ*δ* = *δ*_∥_ − *δ*_⊥_) in eqn (12) is set to 0.12
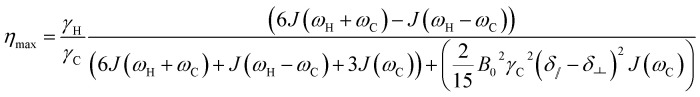


On the basis of the best parameters derived from fitting the relaxation data (Table 2D, CD type spectral density function and CSA contribution) the dependence of *η*_max_ on *τ*_c_ was calculated and depicted in Fig. 3. For comparison also the *η*_max_ dependence on *τ*_c_ was calculated using the fit parameter given in Tables 1A and D (see Fig. S5 and S6†). Because for the selected temperatures (278.2 K, 293.2 K and 323.2 K) the molecular motion of [C_1_C_1_IM][(CH_3_)_2_PO_4_] is not in the fast motion limit (ω_C_*τ*_c_ ≪ 1) and the relaxation by CSA is considered as a leakage term the maximum *η* observable of 1.98 for pure ^1^H–^13^C dipolar interaction can not be reached.

However, an increase in temperature resulting in shorter *τ*_c_ correlates with increased *η* values as shown in Fig. 3. For carbon C2 and C4/5 the observed enhancement factors *η* match the theoretical ones reasonably well and higher enhancements are obtained in the motion regime of *ω*_*C*_*τ*_c_ ≈ 1 at 9.35 T compared with 16.45 T. For the methyl carbons the observed enhancement factors deviate substantially from the expected values. The reason could be that the Δ*δ* values for the methyl groups (Δ*δ* = 267 and 303 ppm, respectively) are overestimated in the fitting procedure. Recalculating the dependence of *η*_max_ on *τ*_c_ with a Δ*δ* value of 100 ppm reveal a nearly perfect agreement between measured and expected NOE enhancement factors (Fig. 3b, dashed lines). This confirms that the CD spectral density function and fitted parameters *β, E*_VFT_*, T*_0_ and *τ*_0_ at least are suitable to model the molecular mobility of [C_1_C_1_IM][(CH_3_)_2_PO_4_]. From fitting the relaxation data and comparing the measured and calculated *η* values the ^13^C relaxation of [C_1_C_1_IM][(CH_3_)_2_PO_4_] partially by CSA can't be excluded. Moreover, for Δ*δ* ≈ 100 ppm the maximum relaxation rate due to CSA is 0.4 s^−1^ (at 9.35 T) and 0.7 s^−1^ (at 16.45 T). CSA contributes field strength dependent to the overall ^13^C relaxation (6% and 17% at 9.35 T and 16.45 T, respectively) which is nevertheless dominantly driven by dipolar ^1^H–^13^C relaxation. However, the absolute Δ*δ* values, mainly for the methyl groups, needs to be taken with caution and it has to be mentioned that the CSA magnitude can vary with temperature. Additional relaxation data at other magnetic field strengths or the direct measurement of the CSA is necessary to verify the fitted Δ*δ* values.

### Diffusion study

To gain a deeper understanding of cation–anion association/dissociation, formation of ion pairs or the aggregation of ions is of vital interest not only in the description of the IL electrical conductance and mass transport but also to rationalise the individual interaction of cation and anion with potential solute molecules.

**Fig. 4:**
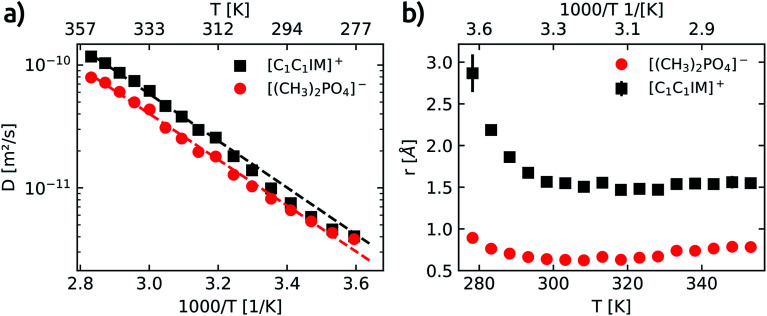
(a) Temperature dependence of the self-diffusion coefficients (D) for the [C_1_C_1_IM]^+^ cation (black squares) and the [(CH_3_)_2_PO_4_]^−^ anion (red circles). Dashed lines show fits for [C_1_C_1_IM]^+^ (black) and [(CH_3_)_2_PO4]^−^ (red) calculated with the Arrhenius equation *D* = *D*_0_ exp(−*E*_A_/*RT*). *D*_0_ is a fitting constant, *E*_a_ gives the activation energy for self-diffusion, *R* is the universal gas constant and *T* is the temperature. (b) Calculated effective hydrodynamic radii (*r*) of [C_1_C_1_IM]^+^ (black squares) and [(CH_3_)_2_PO_4_]^−^ (red circles) as a function of temperature.

Each of the four well resolved resonances in the ^1^H spectrum of [C_1_C_1_IM][(CH_3_)_2_PO_4_] has been used to determine the temperature dependence of the translational self-diffusion coefficients (*D*_t_). Three out of the four resonances were assigned to the [C_1_C_1_IM]^+^ cation and the *D* values obtained for the cation are averaged (see Table S6†). The temperature dependence of *D* is shown in Fig. 4a and fitted to an Arrhenius-type equation (eqn (10)). The activation energies of translational diffusion, *E*_A_, for the neat [C_1_C_1_IM][(CH_3_)_2_PO_4_] are 36.5 ± 1.9 kJ mol^−1^ and 35.8 ± 2.2 kJ mol^−1^ for the [C_1_C_1_IM]^+^ cation and the [(CH_3_)_2_PO_4_]^−^ anion, respectively. These values are in the same order found for other ILs.^59–61,74–76^ It is interesting that *E*_A_ for diffusion and rotational correlation give nearly the same values at least for the cation. The activation energy of the anion for diffusion is slightly higher compared to the rotational correlation.

The increase in the diffusion coefficients for both cation and anion with increasing temperature is to be expected. The diffusion coefficients associated with the [C_1_C_1_IM]^+^ cation are higher than the values obtained for the [(CH_3_)_2_PO_4_]^−^ for the whole temperature range. The apparent cationic transference number and the predicted molar conductivity derived from the self-diffusion coefficients of [C_1_C_1_IM][(CH_3_)_2_PO_4_] are calculated and plotted in Fig. S7.† In contrast to other imidazolium based ILs,^59–61^ here, we observe an increase in the apparent cationic transference number with increasing temperature (Fig. S7a†). This can be rationalised by the slightly lower activation energy for the diffusion of the anion compared to the cation. At higher temperatures the diffusion of [(CH_3_)_2_PO_4_]^−^ increases relatively less compared to the IL cation. The high cationic transference numbers of [C_1_C_1_IM][(CH_3_)_2_PO_4_] also reveal a faster diffusion of the cation than the anion, even though both ions are similar in size, and that the difference in cationic and anionic diffusion increases with temperature.

As already reported in earlier studies on several imidazolium^59–61,74,75^ or piperidinium^76^ based ILs also in our investigation the anion diffuses at a slower rate than the cation. In most of these previous reports the slower diffusing anion was smaller compared to a larger IL cation. The primary aggregation of IL anions resulting in a lower anionic diffusion constant was proposed to rationalise this surprising observation on the one hand.^74,77^ On the other hand it was suggested that the diffusion of the IL cation is faster than expected.^75^

According to the Stokes–Einstein equation (eqn (13)) the self-diffusion coefficient is inversely proportional to hydrodynamic radius (*r*) and, therefore, to the volume (*V*) of a spherical particle under investigation.^78^ For considering the anion and the cation it follow the ratios: ^3^√*V*_cation_/^3^√*V*_anion_ = *r*_cation_/*r*_anion_ = *D*_anion_/*D*_cation_. The calculated van-der-Waals volumes^79^ (and effective radii^74,80^) of the [C_1_C_1_IM]^+^ cation and the [(CH_3_)_2_PO_4_]^−^ anion are 89.8 Å^3^ (2.59 Å) and 96.8 Å^3^ (2.82 Å), respectively, and, hence, result in a theoretical ratio of the diffusion coefficients of *D*_anion_/*D*_cation_ = 0.97 and hydrodynamic radii of *r*_cation_/*r*_anion_ = 0.92 for [C_1_C_1_IM][(CH_3_)_2_PO_4_]. The experimental ratio *D*_anion_/*D*_cation_ continuously decreases from 0.95 at 278.2 K to 0.67 at 353.2 K. At lower temperatures the experimental *D*_anion_/*D*_cation_ is in total agreement with the theoretical ratio and the lower *D*_anion_ can be explained by the larger anionic volume. However, at higher temperatures the experimental *D*_anion_/*D*_cation_ deviates clearly from the theoretical ratio.13
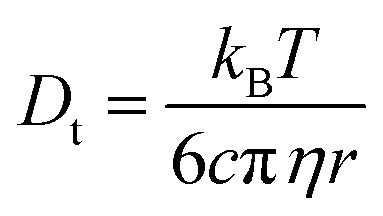
In order to clarify the disparity the hydrodynamic radii of [C_1_C_1_IM]^+^ and [(CH_3_)_2_PO_4_]^−^ were evaluated on the basis of the experimental data. The Stokes–Einstein relationship (eqn (13)), where *k*_B_ and *T* correspond to Boltzmann constant and the temperature, models the self-diffusion coefficient (*D*_t_) for a sphere of an effective hydrodynamic radius (*r*) with the temperature depenent viscosity (*η*) of a solution. Here, *c* is a constant that depends on the hydrodynamic particle-fluid boundary conditions. Assuming infinite dilution of a large diffusing sphere compared to the surrounding solvent the constant *c* = 1 (stick boundary conditions). In situations where solute and solvent are of similar size (slip boundary conditions) or intermolecular interaction, non-spherical shape or aggregation need to be considered in the adjustment of the constant *c* was proposed.^18,74,80–82^ In our analysis the value 2/3 is applied for the constant *c*.

In a classical description the rotational correlation time *τ*_c_ of an isotropically diffusing sphere is given by eqn (14).^18,78,81,82^ The combination of eqn (13) and (14) by eliminating *η, k*_B_ and *T* makes it possible to correlate both NMR accessible values *D*_t_ and *τ*_c_ to the hydrodynamic radius *r* of a particle under investigation (eqn (15)). Applying the fit parameters from Table 2D allowed the calculation of *τ*_c_ for every temperature.14
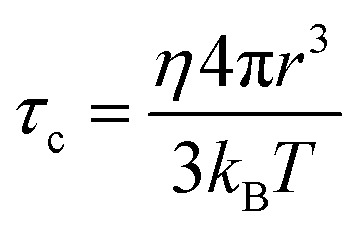
15
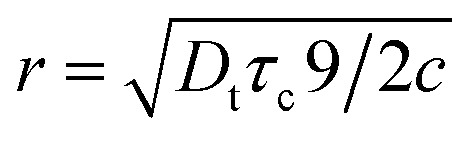
Based on eqn (15) the hydrodynamic radii of the [C_1_C_1_IM]^+^ cation and [(CH_3_)_2_PO_4_]^−^ anion were calculated and presented in Fig. 4b as a function of temperature. The hydrodynamic radius of the anion slightly decrease in the temperature range between 278–293 K and is then nearly constant for the temperature range studied. In contrast the hydrodynamic radius of the cation significantly reduces with increasing temperature up to 293 K and then reaches a constant value at higher temperatures. The simultaneous decrease in the hydrodynamic radii of both the cation and the anion between 278–293 K suggests the formation of cationic–anion pairs. However, it is reasonable to draw the conclusion that no long existing cation–anion ion pairs are formed in the temperature range above 293 K. Averaged over the temperature range above 293 K the hydrodynamic radii of the [C_1_C_1_IM]^+^ cation and the [(CH_3_)_2_PO_4_]^−^ anion are 1.73 ± 0.06 Å and 0.71 ± 0.07 Å, respectively. By setting the constant *c* to 1 the obtained hydrodynamic radii are slightly larger ([C_1_C_1_IM]^+^: *r* = 1.87 ± 0.04 Å, [(CH_3_)_2_PO_4_]^−^: *r* = 0.87 ± 0.09 Å). In addition, the ^13^C correlation times were plotted *versus* the inverse of the diffusion coefficient (see Fig. S8†). The slopes also provide information about *r*. The hydrodynamic radii of [C_1_C_1_IM]^+^ and [(CH_3_)_2_PO_4_]^−^ obtained from this correlation are 1.64 ± 0.03 Å (2.00 ± 0.03 Å for *c* = 1) and 0.72 ± 0.02 Å (0.88 ± 0.02 Å for *c* = 1), respectively. Huang *et al.* correctly point out the limitations (isotropic reorientation of a spherical-top) of hydrodynamic radii derived from reorientation dynamics.^68^ Nevertheless, for small and symmetric particles the applied theory is appropriate.

The experimentally acquired *r* of the cation is slightly smaller than theoretically estimated but in good agreement with other results for imidazolium based IL cations.^68,74,82,83^ Within limits, this corroborates the Stokes–Einstein approximation made and shows the applicability of eqn (15) for estimating hydrodynamic radii based on *τ*_c_ and *D* at least for the imidazolium based IL cation.

However, the experimentally based radius of the anion seems to be unrealistic small and deviates significantly from the theoretical value. If the aforementioned consideration (*r*_cation_/*r*_anion_ = *D*_anion_/*D*_cation_) holds true, one should expect nearly the same value for both ratios. The experimental ratio of the hydrodynamic radii *r*_cation_/*r*_anion_ is in the range of 1.98–2.52 and reveals a clear contradiction to the experimentally obtained ratio of the diffusion coefficients *D*_anion_/*D*_cation_ = 0.76 averaged over the temperature range.

Assuming the radius of the anion derived from rotational correlation times, and hence, the ratio *r*_cation_/*r*_anion_ = 1.98–2.52, is correct, this would imply that the measured diffusion coefficients of the [(CH_3_)_2_PO_4_]^−^ anion are too small by a factor of ≈2.6–3.3. However, it is more likely that the calculated radius of the anion based on eqn (15) is too small. Under the premise that the calculated *τ*_c_ values of the anion are correct and that the actual hydrodynamic radius of a single and isolated [(CH_3_)_2_PO_4_]^−^ particle has nearly the same value as the cation (≈2 Å, s. above) the measured diffusion coefficients of the anion are too small by a factor of ≈4 to 6 for this molecular size and *τ*_c_ values. In reverse, if it means that in average 4–6 [(CH_3_)_2_PO_4_]^−^ anions cluster, is subject to speculation. The [(CH_3_)_2_PO_4_]^−^ anion diffuse substantially slower than expected, in particular in the high temperature range. To explain these phenomena the concept of anion-rich aggregates in ILs was recently suggested and experimentally verified.^74,77^ Along this line, the diffusion coefficients and, thus, the derived hydrodynamic radii of [C_1_C_1_IM]^+^ and [(CH_3_)_2_PO_4_]^−^ and their interpretation presented in this study render the formation of anionic aggregates a suitable model to explain the comparatively low diffusion coefficients of the anion. One should keep in mind the different NMR time scales at which the diffusion (ms to s) and the reorientation (ns) dynamics are studied. Both time scales are too long for the detection of short-living uncharged IL ion pairs. The lifetime of such ion pairs was estimated to be in ≥ps time frame.^18^ The longitudinal ^13^C relaxation of [C_1_C_1_IM][(CH_3_)_2_PO_4_] may reflects the rotational reorientation of single/not aggregated ionic particles in the ns time regime whereas the diffusion represents a “longtime” averaged clustering/aggregation mainly of the anions. Based on our NMR data presented here no reliable statement about the number of clustering anions is possible. The concept of cooperative hydrogen bonding was introduced to rationalise the cationic aggregates.^84,85^ The question needs to be resolved how anionic aggregates can overcome the repulsive Coulomb interaction.

## Conclusion

Here, we report about the analysis of reorientation mobility of the room temperature IL [C_1_C_1_IM][(CH_3_)_2_PO_4_] on the basis of ^13^C longitudinal relaxation times measured at two magnetic field strengths by means of standard NMR liquid probes and by applying magic angle spinning. In addition, an analysis of the diffusion coefficients measured by PFG-NMR techniques at temperatures ranging from 278 K to 353 K was performed to correlate the rotational correlation times and the diffusion coefficients with the hydrodynamic radii of the [C_1_C_1_IM]^+^ cation and the [(CH_3_)_2_PO_4_]^−^ anion.

Despite their high viscosity pure imidazolium based ILs with a melting point at ambient temperatures can be properly studied by standard liquid NMR probe heads. With respect to IL signal line width, and hence resolution no improvement could be obtained by applying HR-MAS probes.

Therefore, the ^13^C longitudinal relaxation behaviour of the [C_1_C_1_IM]^+^ cation as well as the [(CH_3_)_2_PO_4_]^−^ anion within the tested temperature range is reliably described by the BPP theory, the application of the generalised order parameter and an Arrhenius type *τ*_c_ temperature dependence. However, with regard to the goodness-of-fit (*χ*_red_^2^ in Tables 1 and 2) the ^13^C longitudinal relaxation behaviour is more precisely represented by the application of the CD type spectral density function, VFT type *τ*_c_ temperature dependence and the consideration of CSA to ^13^C relaxation. This agrees with the findings from others.^23,47^ Additional relaxation data at a wider range of magnet field strengths and/or temperatures would be necessary to corroborate the distinction between different dynamic models and the total amount of CSA contribution. In this respect, it has to be mentioned that field-cycling NMR relaxometry is a valuable technique to obtain information on molecular dynamics over a broad range of Larmor frequencies (kHz to MHz).^48,86^ We wish to point out that field-cycling NMR relaxometry is successfully employed to investigate the dynamics of ILs recently.^26,45,47,63,87–92^

Although from a theoretical point of view similar in size, the [C_1_C_1_IM]^+^ cation and the [(CH_3_)_2_PO_4_]^−^ anion reveal different reorientation mobilities and diffusivities. The [(CH_3_)_2_PO_4_]^−^ anion shows a three to five times faster reorientation at room temperature compared to the cation. This indicates that cation and anion are not tightly associated in their reorientation mobility.

The temperature dependence of the self-diffusion coefficients is sufficiently described by the Arrhenius equation in the selected temperature range. In the course of the diffusion measurements we did not observe any indications of phase separation, and hence structural heterogeneity. It can be speculated that at lower temperatures the translational diffusion don't follow an Arrhenius-type but rather a VFT-type thermally activated process, as already observed for ILs.^47,93^ Intriguingly, the activation energies derived from relaxation data and diffusion measurements are nearly the same at least for the cation. This suggests that the effective size of the [C_1_C_1_IM]^+^ cation is the same for rotational correlation and diffusion. For the [C_1_C_1_IM]^+^ cation the hydrodynamic radius derived from rotational correlation times and diffusion coefficients fits very well with theoretical considerations and imply the existence of single dissociated cationic particles.

Assuming that the hydrodynamic radius of an isolated [(CH_3_)_2_PO_4_]^−^ anion is similar to the [C_1_C_1_IM]^+^ cation the measured diffusion coefficients of the anion are too small to corroborate the model of single diffusing anionic entities. In contrast, the [(CH_3_)_2_PO_4_]^−^ anion diffuses slower than expected and reveals a diffusion behaviour that indicates the formation of anionic aggregates.

Mainly with respect to IL ions interacting with solute molecules a better understanding of the ionic aggregation state and the dissociated action of IL cation and anion will help to rationalise the effects observed.

It has to be proven further whether and by which way of action the presence of solute molecules (in addition to carbohydrates *e.g.* peptides or proteins) in pure ILs has a measurable impact on the IL microstructure.

## Conflicts of interest

There are no conflicts to declare.

## Supplementary Material

RA-009-C9RA07731F-s001
