# Supplementary material for: Reorientation dynamics and ion diffusivity of neat dimethylimidazolium dimethylphosphate probed by NMR spectroscopy
Source: RSC Adv. 2019 Nov 4;9(61):35735–50. doi: 10.1039/c9ra07731f (PMC9074696; doi:10.1039/c9ra07731f)
Supplement: RA-009-C9RA07731F-s001 [file RA-009-C9RA07731F-s001.pdf]

Electronic Supplementary Material (ESI) for RSC Advances.
This journal is © The Royal Society of Chemistry 2019

Reorientation dynamics and ion diffusivity of neat dimethylimidazolium dimethylphosphate probed by NMR spectroscopy

Christoph Wiedemann[1,*], Günter Hempel[2] and Frank Bordusa[1,*]
[1]Institute of Biochemistry and Biotechnology, Charles Tanford Protein Center, Martin Luther University Halle-Wittenberg, Kurt-Mothes-Str. 3a, D-06120 Halle (Saale), Germany
[2]Institute of Physics, Martin Luther University Halle-Wittenberg, Betty-Heimann-Str. 7, D-06120 Halle (Saale), Germany
[*]Corresponding author: christoph.wiedemann@biochemtech.uni-halle.de, frank.bordusa@biochemtech.uni-halle.de

Supplementary Material

^{1}H and ^{13}C NMR spectrum of $[C_1C_1IM][(CH_3)_2PO_4]$

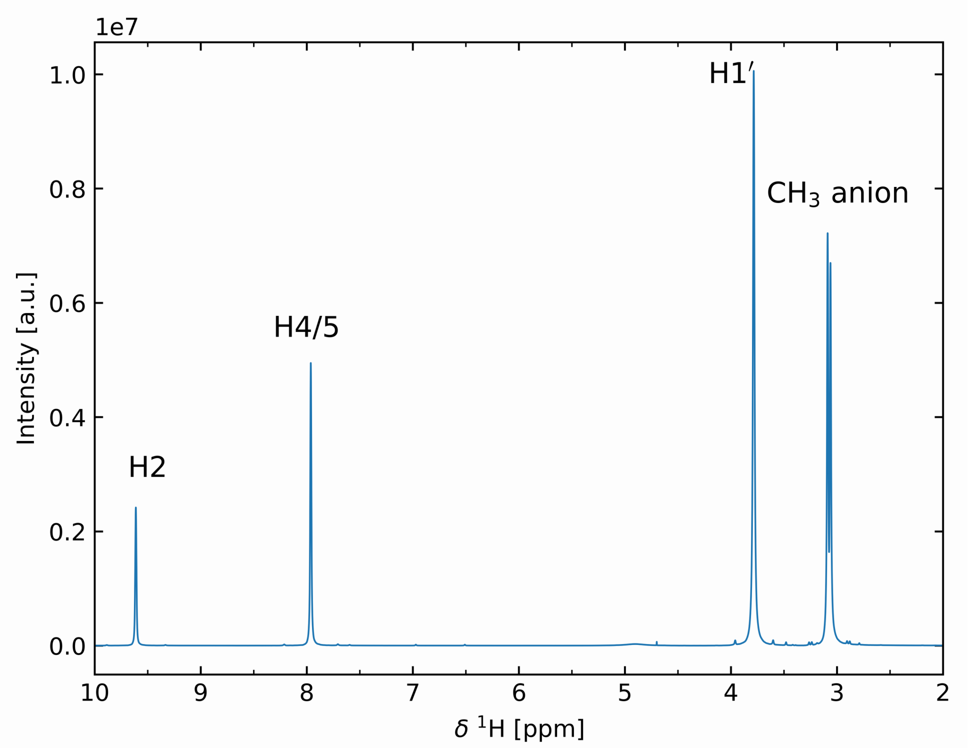

Figure S1: ^{1}H NMR spectrum of pure $[C_1C_1IM][(CH_3)_2PO_4]$ measured at 293 K and a magnetic field strength of 16.45 T. Atom numbering as reported in Fig. 1 (s. main text)

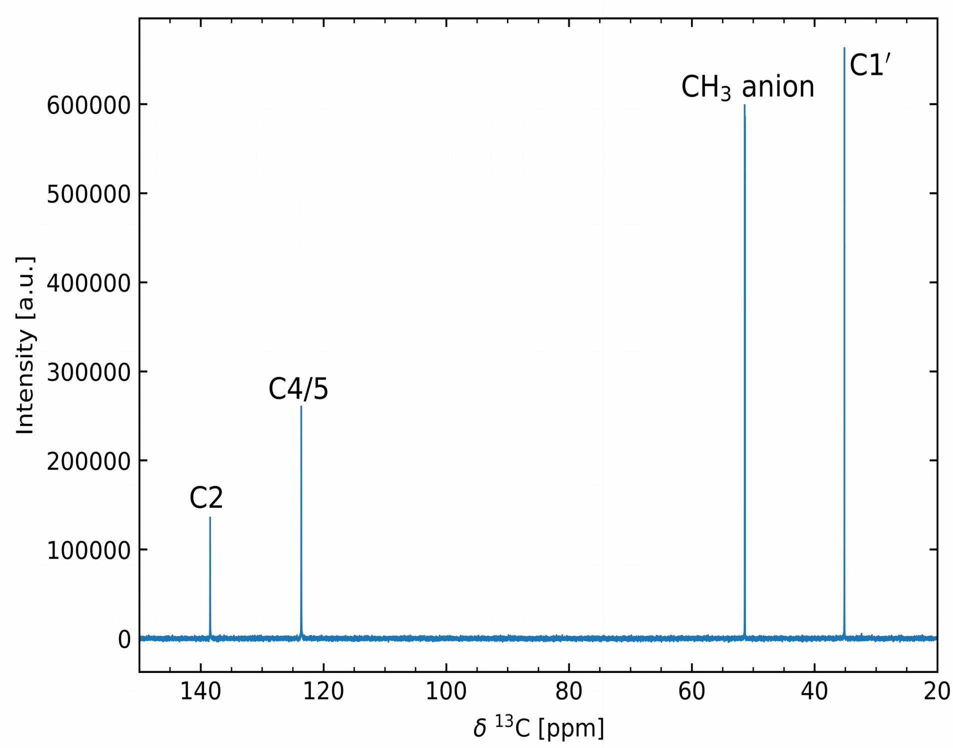

Figure S2: ^{13}C NMR spectrum of pure $[C_1C_1IM][(CH_3)_2PO_4]$ measured at 293 K and a magnetic field strength of 16.45 T. Atom numbering as reported in Fig. 1 (s. main text)

Comparison of ^{13}C T_1 values and line widths measured with HR-MAS and liquid probes

Table S1: Comparison of the ^{13}C T_1 values (s) of $[C_1C_1IM][(CH_3)_2PO_4]$ between broadband and gated ^{1}H decoupling at different temperatures, magnetic field strengths (9.35 T and 16.45 T corresponding to a ^{13}C resonance frequency of 100.6 MHz and 176.2 MHz, respectively) and probe settings.

	100 MHz		176 MHz	
	Broadband/Gated decouple		Broadband/Gated decouple	
	HR-MAS probe	liquid probe	HR-MAS probe	liquid probe
C2				
278.2 K	0.29/0.30	0.31/0.32	0.52/0.49	0.51/0.51
293.2 K	0.23/0.23	0.23/0.23	0.36/0.37	0.35/0.38
323.2 K	0.36/0.36	0.38/0.40	0.53/0.61	0.45/0.47
C4/5				
278.2 K	0.29/0.28	0.30/0.30	0.47/0.46	0.49/0.47
293.2 K	0.21/0.22	0.22/0.22	0.33/0.34	0.34/0.35
323.2 K	0.31/0.33	0.35/0.36	0.49/0.55	0.40/0.42
C1'				
278.2 K	1.19/1.24	1.29/1.28	1.75/1.65	1.82/1.75
293.2 K	0.87/0.94	0.89/0.89	1.33/1.17	1.29/1.27
323.2 K	1.04/1.10	1.21/1.20	1.41/1.48	1.37/1.28
CH_3 anion				
278.2 K	1.30/1.37	1.37/1.33	1.65/1.67	1.80/1.87
293.2 K	1.53/1.54	1.50/1.52	1.84/1.87	1.80/1.87
323.2 K	2.62/2.62	3.07/3.04	3.56/3.81	3.01/3.02

Table S2: The line widths of $[C_1C_1IM][(CH_3)_2PO_4]$ ^{13}C signals at half peak height at 278.2 K, 293.2 K and 323.2 K for different probe types at B_0 of 9.35 T and 16.45 T, respectively (^{13}C resonance frequency of 100.6 MHz and 176.2 MHz). Line widths are given in Hz and the errors are less than 1 Hz.

	100 MHz		176 MHz	
	HR-MAS probe	liquid probe	HR-MAS probe	liquid probe
C2				
278.2 K	7	7	7	7
293.2 K	3	3	4	5
323.2 K	2	2	3	2
C4/5				
278.2 K	7	7	7	7
293.2 K	3	3	3	3
323.2 K	2	2	3	2
C1'				
278.2 K	2	2	3	2
293.2 K	1	1	1	2
323.2 K	1	1	3	1
CH_3 anion				
278.2 K	1	2	2	2

	100 MHz		176 MHz	
	HR-MAS probe	liquid probe	HR-MAS probe	liquid probe
293.2 K	1	1	1	1
323.2 K	1	1	1	1

Longitudinal ^{13}C-relaxation times

Table S3: ^{13}C T1 relaxation times [s] of different carbon groups in $[C_1C_1IM][(CH_3)_2PO_4]$ at B_0 of 9.35 T and 16.45 T

	C2		C4/5		C1'		CH_3 anion	
Temperature [K]	100 MHz	176 MHz	100 MHz	176 MHz	100 MHz	176 MHz	100 MHz	176 MHz
278.2	0.309	0.519	0.296	0.492	1.217	1.925	1.277	1.780
283.2	0.267	0.445	0.254	0.412	1.075	1.607	1.370	1.703
288.2	0.241	0.393	0.226	0.361	0.970	1.365	1.440	1.695
293.2	0.231	0.359	0.215	0.337	0.895	1.330	1.535	1.895
298.3	0.231	0.353	0.213	0.315	0.892	1.240	1.700	1.963
303.2	0.249	0.341	0.229	0.305	0.911	1.200	1.970	2.140
308.2	0.272	0.349	0.251	0.315	0.959	1.335	2.180	2.410
313.2	0.302	0.397	0.278	0.371	1.045	1.350	2.425	2.765
318.2	0.340	0.422	0.312	0.398	1.130	1.400	2.795	2.905
323.2	0.387	0.457	0.355	0.410	1.275	1.495	3.185	3.083
328.2	0.429	0.493	0.389	0.442	1.315	1.570	3.410	3.170
333.2	0.489	0.560	0.446	0.505	1.460	1.600	3.760	3.840
338.2	0.560	0.626	0.505	0.562	1.625	1.770	4.190	4.170
343.2	0.642	0.729	0.578	0.657	1.797	2.025	4.623	4.770
348.2	0.730	0.815	0.655	0.727	1.970	2.210	4.967	5.235
353.2	0.837	0.894	0.753	0.782	2.183	2.290	5.517	5.455

Calculation of τ_c and $E_{A/VFT}$

Table S4: ***A)*** *S^2 calculated in the ^{13}C T_1 minimum according to $S^2=\omega_C/(1.87\ A_0\ T_1^{min})$ with the maximum point of ^{13}C relaxation at $\omega\tau_c=0.791$ (BPP spectral density function was used). τ_c were calculated as proposed in Matveev et al.[1]* ***B)*** *Arrhenius approximation of the τ_c* temperature dependence. ***C)*** *Vogel-Fulcher-Tammann approximation of the τ_c* temperature dependence. [a]*Fast methyl-group rotation was considered by modifying S^2 with the factor 0.11 ($S^2=0.11S_{Met}^2$). For methyl carbons S_{Met}^2 is given. Values for relaxation data at 9.35 T and 16.45 T, respectively.* [b]*Fitted S^2 with a C-H bond length of 1.13* Å

		S^2	τ_c at 298 K (ns)
**A)**	C2	0.68(0.84[b])/0.81(0.99[b])	1.22/0.98
	C4/5	0.74/0.90	1.22/0.97
	C1'	0.55[a]/0.72[a]	1.22/0.98
	CH_3 anion	-/0.45[a]	-/0.31

		E_A (kJ mol^{-1})	τ_0 (s)	χ^2_{red}
**B)**	C2	35.29/34.83	4.76e^{-16b}/4.74e^{-16}	3.14/2.61
	C4/5	34.56/30.04	6.31e^{-16}/2.61e^{-15}	4.39/11.35
	C1'	32.52/27.34	1.89e^{-15}/9.59e^{-15}	3.21/11.81
	CH_3 anion	-/21.50	-/3.69e^{-14}	-/9.75

		E_{VFT} (kJ mol^{-1})	T_0 (K)	τ_0 (s)	χ^2_{red}
**C)**	C2	3.04/2.91	230.61/237.89	4.45e^{-12}/3.29e^{-12}	16.11/48.34
	C4/5	3.02/6.55	230.06/189.78	4.76e^{-12}/5.54e^{-13}	12.93/6.55
	C1'	2.01/3.68	245.35/217.10	1.46e^{-11}/3.79e^{-12}	39.05/12.00
	CH_3 anion	-/4.60	-/188.44	-/1.84e^{-12}	-/5.66

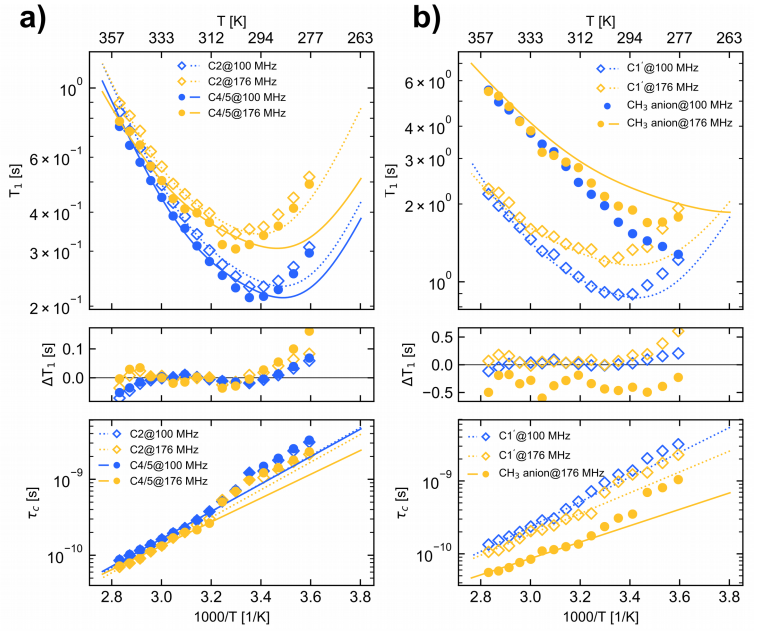

*Figure S3: ^{13}C longitudinal relaxation times (upper panels), the difference between measured and fitted relaxation times (middle panel) and the calculated τ_c values (lower panel) for carbons in $[C_1C_1IM][(CH_3)_2PO_4]$ as a function of temperature. (**a**) carbon C2 (open squares and dotted lines) and C4/5 (filled circles and solid lines), (**b**) carbon C1' (open squares and dotted lines) and CH_3 carbons of the anion (filled circles and solid lines). For denotation see Fig. 1. Blue and yellow markers represent data measured at B_0 of 9.35 T and 16.45 T corresponding to a ^{13}C resonance frequency of 100.6 MHz and 176.2 MHz, respectively. The lines in the upper panel represent calculated ^{13}C relaxation times according the fit parameters given in Tab. S4B. Although no obvious deviation of τ_c from the Arrhenius behaviour is visible for all carbons in the applied temperature range, only the range from 313 K to 353 K was used for the Arrhenius approximation.*

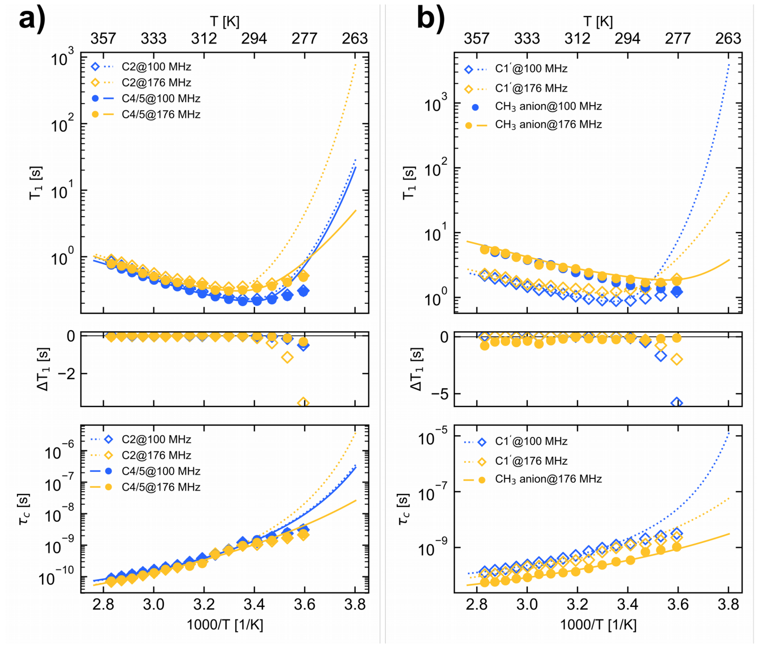

*Figure S4: ^{13}C longitudinal relaxation times (upper panels), the difference between measured and fitted relaxation times (middle panel) and the calculated τ_c values (lower panel) for carbons in $[C_1C_1IM][(CH_3)_2PO_4]$ as a function of temperature. (**a**) carbon C2 (open squares and dotted lines) and C4/5 (filled circles and solid lines), (**b**) carbon C1' (open squares and dotted lines) and CH_3 carbons of the anion (filled circles and solid lines). For denotation see Fig. 1. Blue and yellow markers represent data measured at B_0 of 9.35 T and 16.45 T corresponding to a ^{13}C resonance frequency of 100.6 MHz and 176.2 MHz, respectively. The lines in the upper panel represent calculated ^{13}C relaxation times according the fit parameters given in Tab. S4C.*

$\{^1H\}$-^{13}C NOE enhancement

Table S5: Comparison of $\{^1H\}$-^{13}C NOE enhancement η of $[C_1C_1IM][(CH_3)_2PO_4]$ at different temperatures, magnetic field strengths (9.35 T and 16.45 T corresponding to a ^{13}C resonance frequency of 100.6 MHz and 176.2 MHz, respectively) and probe settings. Spinning rate for HR-MAS conditions was 6 kHz.

	100 MHz		176 MHz	
	HR-MAS probe	liquid probe	HR-MAS probe	liquid probe
C2				
278.2 K	0.47	0.32	0.26	0.30
293.2 K	0.48	0.46	0.31	0.26
323.2 K	1.01	1.32	1.06	0.84
C4/5				
278.2 K	0.31	0.31	0.41	0.24
293.2 K	0.58	0.43	0.39	0.28
323.2 K	1.08	1.35	1.13	0.77
C1'				
278.2 K	0.58	0.50	0.67	0.50
293.2 K	0.55	0.57	0.49	0.48
323.2 K	1.46	1.21	1.04	0.83
CH_3 anion				
278.2 K	0.89	0.69	0.76	0.59
293.2 K	0.84	0.90	0.73	0.67
323.2 K	1.38	1.45	1.13	1.17

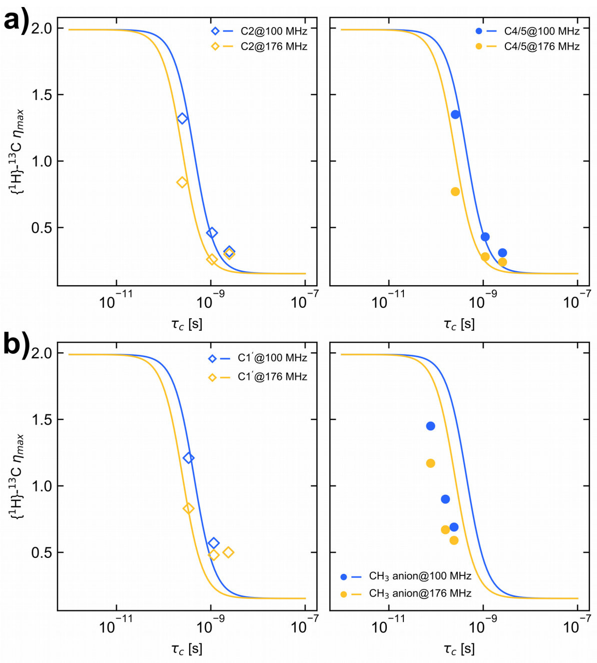

*Figure S5: Dependence of maximum $\{^1H\}$-^{13}C NOE enhancement (η_{max}) on τ_c calculated for a BPP spectral density function with the fit parameters given in Tab. 1A (solid lines). (**a**) carbon C2 (left, open squares) and C4/5 (right, filled circles), (**b**) carbon C1' (left, open squares) and CH_3 carbon of the anion (right, filled circles). Blue and yellow markers represent data measured at B_0 of 9.35 T and 16.45 T corresponding to a ^{13}C resonance frequency of 100.6 MHz and 176.2 MHz, respectively.*

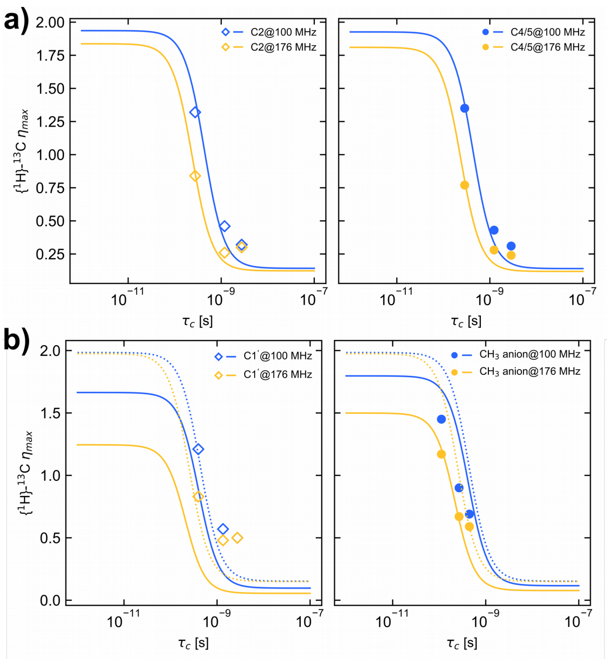

*Figure S6: Dependence of maximum $\{^1H\}$-^{13}C NOE enhancement (η_{max}) on τ_c calculated for a BPP spectral density function and considering CSA with the fit parameters given in Tab. 2A (solid lines). (**a**) carbon C2 (left, open squares) and C4/5 (right, filled circles), (**b**) carbon C1' (left, open squares) and CH_3 carbon of the anion (right, filled circles). Dashed lines in **b)** are calculated with the fit parameters given in Tab. 2A but a CSA value of 30 ppm. Blue and yellow markers represent data measured at B_0 of 9.35 T and 16.45 T corresponding to a ^{13}C resonance frequency of 100.6 MHz and 176.2 MHz, respectively.*

Diffusion study

Table S6: Self-diffusion coefficients of $[C_1C_1IM][(CH_3)_2PO_4]$ as a function of temperature

	D (m^2/s)				
	C2	C4/5	C1'	Ø $[C_1C_1IM]^+$	anion CH_3
278.2 K	3.93e-12	4.07e-12	4.09e-12	4.03±0.07e-12	3.83e-12
283.2 K	4.59e-12	4.58e-12	4.59e-12	4.58±0.01e-12	4.31e-12
288.2 K	5.77e-12	5.85e-12	5.76e-12	5.79±0.04e-12	5.35e-12
293.2 K	7.51e-12	7.53e-12	7.49e-12	7.51±0.02e-12	6.59e-12
298.2 K	9.90e-12	9.92e-12	9.87e-12	9.89±0.02e-12	8.18e-12
303.2 K	1.40e-11	1.38e-11	1.39e-11	1.39±0.01e-11	1.03e-11
308.2 K	1.81e-11	1.82e-11	1.80e-11	1.81±0.01e-11	1.28e-11
313.2 K	2.58e-11	2.58e-11	2.54e-11	2.57±0.02e-11	1.80e-11
318.2 K	2.98e-11	2.99e-11	2.93e-11	2.97±0.03e-11	1.96e-11
323.2 K	3.80e-11	3.84e-11	3.78e-11	3.81±0.03e-11	2.52e-11
328.2 K	4.68e-11	4.65e-11	4.59e-11	4.64±0.04e-11	3.09e-11
333.2 K	6.26e-11	6.03e-11	6.19e-11	6.16±0.09e-11	4.35e-11
338.2 K	7.53e-11	7.44e-11	7.27e-11	7.41±0.11e-11	4.97e-11
343.2 K	8.75e-11	8.72e-11	8.50e-11	8.66±0.11e-11	6.05e-11
348.2 K	1.05e-10	1.05e-10	1.01e-10	1.03±0.02e-10	0.72e-10
353.2 K	1.19e-10	1.17e-10	1.16e-10	1.17±0.01e-10	0.79e-10

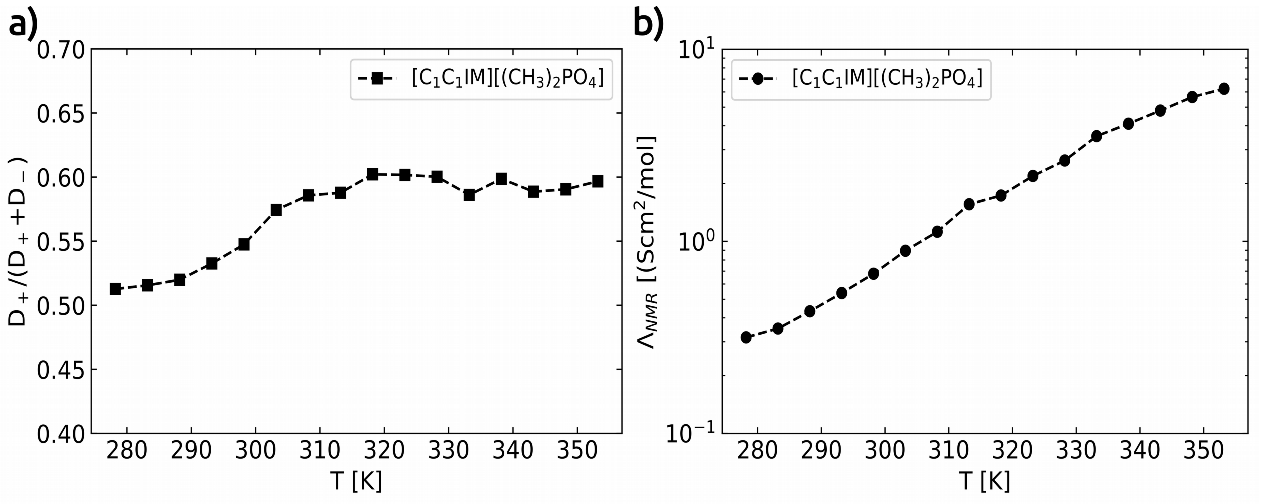

*Figure S7: The apparent cationic transference number (**a**) and the predicted molar conductivity (**b**) derived from the self-diffusion coefficients of $[C_1C_1IM][(CH_3)_2PO_4]$ are plotted as a function of temperature[2–4]. The apparent cationic transfer number is calculated on basis of cationic and anionic diffusion coefficients ($D_+/(D_++D_-)$). The molar molar conductivity, Λ_{NMR}, is calculated according to the Nernst-Einstein equation ($\Lambda_{NMR}=F^2(D_{[C1C1IM]+}+D_{[(CH3)2PO4]-})/RT$, F:Faraday constant, D:cationic and anionic diffusion coefficient, R:universal gas constant, T:temperature) taking account of cationic and anionic diffusion coefficients.*

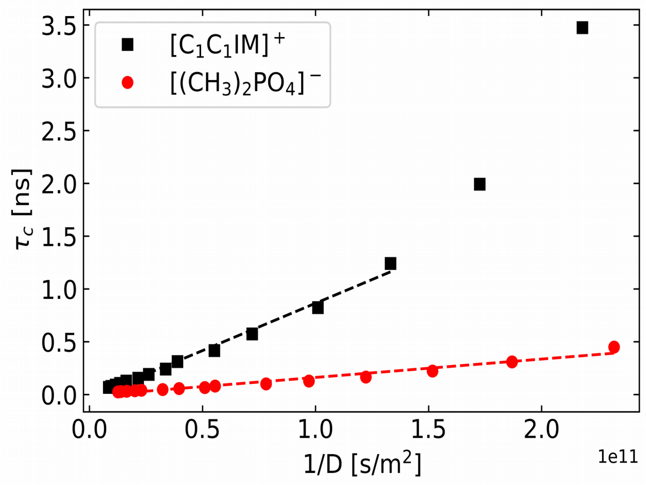

Figure S8: [13]C correlation times τ_c [ns] plotted as function of the inverse self-diffusion coefficients D [s/m^2] for $[C_1C_1IM]^+$ (black squares) and $[(CH_3)_2PO_4]^-$ (red circles) from 278.2 K to 353.2 K. Dashed lines show fits according to $\tau_c=(2r^2/9cD)+\tau_0$. The constant c was set to 2/3 and the slope provides information about the hydrodynamic radius r. For $[C_1C_1IM]^+$ r_{cation}=1.64±0.03 Å and for $[(CH_3)_2PO_4]^-$ r_{anion}=0.72±0.02 Å, respectively.

<u>**References**</u>

1 V. V. Matveev, D. A. Markelov, E. A. Brui, V. I. Chizhik, P. Ingman and E. Lähderanta, 13C NMR relaxation and reorientation dynamics in imidazolium-based ionic liquids: revising interpretation., *Phys. Chem. Chem. Phys.*, 2014, **16**, 10480–4.

2 H. Tokuda, K. Hayamizu, K. Ishii, M. A. B. H. Susan and M. Watanabe, Physicochemical properties and structures of room temperature ionic liquids. 1. Variation of anionic species, *J. Phys. Chem. B*, 2004, **108**, 16593–16600.

3 H. Tokuda, K. Hayamizu, K. Ishii, M. A. B. H. Susan and M. Watanabe, Physicochemical properties and structures of room temperature ionic liquids. 2. variation of alkyl chain length in imidazolium cation, *J. Phys. Chem. B*, 2005, **109**, 6103–6110.

4 H. Tokuda, K. Ishii, M. A. B. H. Susan, S. Tsuzuki, K. Hayamizu and M. Watanabe, Physicochemical properties and structures of room-temperature ionic liquids. 3. Variation of cationic structures, *J. Phys. Chem. B*, 2006, **110**, 2833–2839.
